# Mipu1, a Novel Direct Target Gene, Is Involved in Hypoxia Inducible Factor 1-Mediated Cytoprotection

**DOI:** 10.1371/journal.pone.0082827

**Published:** 2013-12-11

**Authors:** Kangkai Wang, Jian Lei, Jiang Zou, Hui Xiao, Anlan Chen, Xiaoliu Liu, Ying Liu, Lei Jiang, Zihui Xiao, Xianzhong Xiao

**Affiliations:** 1 Department of Pathophysiology, Xiangya School of Medicine, Central South University, Changsha, Hunan, China; 2 Department of Pathology, the Affiliated Tumor Hospital, Xiangya School of Medicine, Central South University, Changsha, Hunan, China; 3 Department of Endocrinology, the Second Xiangya hospital, Xiangya School of Medicine, Central South University, Changsha, Hunan, China; University of Nebraska Medical Center, United States of America

## Abstract

Mipu1 (myocardial ischemic preconditioning up-regulated protein 1), recently identified in our lab, is a novel zinc-finger transcription factor which is up-regulated during ischemic preconditioning. However, it is not clear what transcription factor contributes to its inducible expression. In the present study, we reported that HIF-1 regulates the inducible expression of Mipu1 which is involved in the cytoprotection of HIF-1α against oxidative stress by inhibiting Bax expression. Our results showed that the inducible expression of Mipu1 was associated with the expression and activation of transcription factor HIF-1 as indicated by cobalt chloride (CoCl_2_) treatment, HIF-1α overexpression and knockdown assays. EMSA and luciferase reporter gene assays showed that HIF-1α bound to the hypoxia response element (HRE) within Mipu1 promoter region and promoted its transcription. Moreover, our results revealed that Mipu1 inhibited the expression of Bax, an important pro-apoptosis protein associated with the intrinsic pathway of apoptosis, elevating the cytoprotection of HIF-1 against hydrogen peroxide (H_2_O_2_)-mediated injury in H9C2 cells. Our findings implied that Bax may be a potential target gene of transcription factor Mipu1, and provided a novel insight for understanding the cytoprotection of HIF-1 and new clues for further elucidating the mechanisms by which Mipu1 protects cell against pathological stress.

## Introduction

Hypoxia inducible factor 1 (HIF-1) is expressed ubiquitously in almost all mammalian cells. HIF-1 is a heterodimer composed of an oxygen-labile α subunit and a constitutively expressed β subunit. The activity and subcellular localization of HIF-1α is oxygen-sensitive, increasing in response to hypoxia and decreasing under normoxia. However, the HIF-1β is not affected by hypoxia and is constitutively expressed in the nucleus. Under hypoxia, HIF-1α becomes stabilized, translocates from cytoplasm to nucleus and heterodimerizes with HIF-1β. The complex has transcriptional activity and binds to the hypoxia response elements (HREs) in the regulatory regions of the target genes to induce gene expression [1-4]. 

Increasing evidences have proved that HIF-1α plays cardioprotection by inducing expression of many endogenous protective genes including inducible nitric oxide synthase (iNOS) [5], erythropoietinme (EPO) [6], oxygenase-1 (HO-1) [1,7], adiponectin [8] and vascular endothelial growth factor (VEGF) [9] etc. Data obtained by using genetic (gene silencing and over-expressing) and pharmacological (cobalt chloride and desferroxamine) strategies for stabilizing HIF-α, have suggested that HIF-1α is a mediator of ischemic preconditioning (IPC)-mediated acute and delayed phase cardioprotection via activating pro-survival protein kinase signaling, inhibiting reactive oxygen species generation and increasing adenosine formation [1-4,10]. Recently, it has been demonstrated that HIF-1α orchestrates the body’s (tissues’ or cells’) protective response to hypoxia via the transcriptional activation of up to 200 genes [11]. These genes are regulated directly or indirectly by HIF-1α and are involved in a wide-spectrum of cellular functional events including cell proliferation, differentiation, apoptosis, glucose metabolism, neovascularization, vascular activity and remodeling, inflammation, erythropoiesis and so on [1-4,10,11]. 

Mipu1 (myocardial ischemic preconditioning up-regulated protein 1), a novel zinc-finger protein, was recently identified in our lab [12]. It is termed as Mipu1 because of its up-regulation after myocardial ischemic preconditioning. Mipu1 has an open reading frame of 1 827 bp for encoding 608 amino acids which contains a Krϋppel-associated box (KRAB) domain at the N terminal, 14 successive C2H2 type zinc finger domains at the C terminal and a bipartite nuclear targeting sequence at amino acid residues 193 to 277 [12,13]. Mipu1 is expressed abundantly and predominantly in brain and heart and localizes in the nucleus within cell [14,15]. We have demonstrated that Mipu1 serves as a zinc ion-dependent transcription inhibitor with a putative DNA binding site (5′-TGT**CTTA**TCGAA-3′) [16]. It exhibits cytoprotection against oxidative stress and serum deprival, and inhibits the transcriptional activities of SRE and AP-1 [17]. 

Recently, we evidenced that GC box is essential for regulating the constitutive expression of Mipu1 [18]. However, GC box is neither hypoxia-response nor stress-response elements, implying that other transcription factors binding sites within Mipu1 promoter region might be responsible for its up-regulation during pathological stress (ischemic or hypoxic stress). By searching transcription factor binding sites with “TESS” and MatInspector software, one cAMP-response element binding protein (CREB) binding site and one hypoxia response element (HRE) site were identified. Our previous studies showed that oxidative stress-mediated inducible expression of Mipu1 is partially due to the activation of CREB [19]. 

The HRE site locates in the positions -568 to -547 bp of the rat Mipu1 promoter with a high score (Figure S1 in File S1). However, it is still unknown what roles of HRE site are in inducible expression of Mipu1. Since HRE is associated with transcription factor HIF-1 and responses to hypoxia, we presumed that HIF-1 would regulate the inducible expression of Mipu1, and Mipu1 would subsequently contribute to HIF-1-mediated cytoprotection. Our results proved that HIF-1α bound directly to the promoter of Mipu1 and promoted its expression under hypoxia condition mimicked by cobalt chloride (CoCl_2_). HIF-1α-mediated cytoprotection against oxidative stress was partially due to inducible expression of Mipu1 which inhibited the Bax expression in H9C2 cells. The findings provided a novel insight into the regulatory mechanisms of novel gene Mipu1 expression and cytoprotection of HIF-1. 

## Materials and Methods

### Cell culture

Rat H9C2 cardiomyocyte was purchased from ATCC and maintained in Dulbecco’s modified Eagle’s medium (DMEM) supplemented with 10% (v/v) heat inactivated fetal bovine serum (Invitrogen) and 100 units/ml penicillin, 100 µg/ml streptomycin (Sigma) in a humidified CO_2_ incubator at 37 °C. Hydrogen peroxide (H_2_O_2_) was diluted in Ca^2+^-free PBS and further diluted in culture medium. The cells were grown to 80% confluence and then treated with a final concentration of 0.5 mmol/L H_2_O_2_ or 100 μmol/L CoCl_2_ (Sigma).

### Plasmids and siRNA

pcDNA3.1(+)-Mipu1 eukaryotic plasmid and short hairpin RNA (shRNA) of Mipu1 plasmid (pRNA-u6.1-Mipu1) were constructed in our lab as reported previously [17]. For construction of pcDNA3.1(+)-HIF-1α eukaryotic plasmid, the full length open reading frame of rat HIF-1α gene (NM_024359.1) was generated by PCR. The PCR products were cloned into the unique *KpnⅠ*and *EcoRⅠ* of pcDNA3.1(+) vector as previously described [20]. The siRNA against to HIF-1α (sc-45919) and control siRNA (sc-37007) were purchased from Santa Cruz. Lipofectamine® 2000 and Lipofectamine® RNAiMAX reagents were purchased from Invitrogen. 

### Western Blot Analysis

For Western blot analysis, cells from each group were scraped and collected in a10-ml tube, subsequently washed twice with PBS and resuspended with 5 volumes of lysing buffer (50 mM Tris-HCl, pH 7.5, 250 mM NaCl, 5 mM EDTA, 50 mM NaF, 0.5% Nonidet P-40) supplemented with protease inhibitor cocktail (Santa Cruz). The cell lysate was incubated on ice for 30 min and then centrifuged at 10,000 g for 10 min at 4 °C. The protein content of the supernatant was determined by the Bradford assay (Bio-Rad) and diluted to 1 mg/ml. After adding appropriate 6×SDS loading buffer, equal amounts of protein (20-30 μg) were loaded and separated on 10% SDS-PAGE and then transferred electrophoretically onto nitrocellulose membranes. Blots were blocked with 2% albumin in TBST (20 mM Tris-HCl, pH 8.0, 150 mM NaCl, 0.1% Tween-20) overnight at 4 °C and then probed with rabbit-anti-HIF-1α (Santa Cruz), mouse-anti-β-actin (Sigma) or rabbit-anti-Mipu1 antiserum (produced in our lab as described previously [13-15]), respectively. The immune complexes were visualized with an HRP-conjugated secondary antibody and DAB staining kit (Boster Biological Technology, China). Semi-quantitative analysis was performed by automated densitometry.

### Extraction of total RNA and Quantitative Real-time PCR

Total RNA was extracted using RiboPure™ Kit (Invitrogen, USA) following manufacturer’s instruction. cDNA was prepared from 1 μg total RNA by using High Capacity cDNA Reverse Transcription kit (Invitrogen, USA). qRT-PCR was performed in triplicates in an ABI PRISM 7500HT System with 5% cDNA product, 125 nM of primers, and Fast SYBR Green Master Mix (Applied Biosystems). The primers were listed as following, sense 5′-GAAGTTAGAGTCAAGCCCAGAG -3′ and antisense 5′- CTCAGGTGAGCTTTGTCTAGTG -3′ for HIF-1α (109 bp), sense 5′-ATACTGGAGAGAAGCCCTATCA-3′ and antisense 5′-TTCCCACACAGGTCACATTC-3′ for Mipu1 (124 bp), sense 5′-ACTCCCATTCTTCCACCTTTG-3′ and antisense 5′-CCCTGTTGCTGTAGCCATATT-3′ for GAPDH (105 bp) and sense 5′- CACCAGCTCTGAACAGATCATGA -3′ and antisense 5′- TCAGCCCATCTTCTTCCAGATGGT-3′ for Bax (541 bp). Relative expression of target genes was calculated by the 2^-△△CT^ method as previously mentioned [21]. Final data were described as fold changes against control cells. 

### Fluorescence microscopy

Indirect immunofluorescence assay was performed following the method described previously [22], H9C2 cells were grown in 12-well plate. At 6 h after treatment with CoCl_2_ or at 24 h after HIF-1α transfection, the culture medium was quickly removed, cells were rinsed twice in pre-warmed (37 °C) PBS and then fixed in cold (−20 °C) methanol for 10 min, and soaked three times in cold acetone. Then, the cells were washed, incubated in the presence of blocking solution (30 min, room temperature) and then incubated with rabbit-anti-HIF-1α antibody (1:500, Santa Cruz) for 1 h at room temperature. After washing, FITC (fluorescein isothiocyanate) -conjugated antibodies (Boster, Wuhan, China) were added (1 h, 1:500). Nuclei were stained with Hoechst 33258 (Sigma). The slides were mounted and HIF-1α cellular distribution was analyzed by fluorescence microscopy using an Olympus BX61WI upright microscope.

### Caspase-9 activity assay

To analyze Caspase-9 activity, H_2_O_2_ treated and untreated cells (1×10^6^ cells) were washed twice in prewarmed PBS and resuspended in cell lysis buffer (10 mmol/L HEPES, pH 7.4, 2 mmol/L EDTA, 0.1% CHAPS, 5 mmol/L DTT and protease inhibitor cocktail). Absorbance was read with the microplate reader 595 model at 405 nm according to the manufacturer’s instructions (ApoAlert Caspase-9 Colorimetric Assay, Clontech). Results were expressed as the fold increase in caspase-9 activity over control.

### Flow cytometric analysis

Cells were harvested by trypsinization, washed twice with ice-cold PBS, resuspended in ice-cold PBS, and fixed with 70% ethanol. Then the cells were incubated with 1 mL of PI/Triton X-100 staining solution (0.1% Triton X-100 in PBS, 0.2 mg/mL RNase A, and 10 μg/mL propidium iodide) for 30 min at room temperature. The stained cells were analyzed using a FACScan flow cytometry in combination with BD Lysis II Software (Becton Dickinson). Ten thousand events were analyzed for each sample.

### Lactic Dehydrogenase (LDH) release rate

For the LDH release rate assay, an amount of 0.2 ml culture medium was collected at 3 h after o.5 mmol/L H_2_O_2_ exposure. The reaction velocity was monitored by the decrease in absorbance at 340 nm by using the Spectrophotometer according to LDH kit (Jiancheng, Nanjing, China). The results were expressed as % of LDH released.

### Nuclear extracts preparation and electrophoretic mobility shift assays (EMSA)

Nuclear extracts preparation and EMSA were performed according to the methods described previously in our lab [16]. Briefly, after each treatment, cells were harvested and washed twice with cold PBS. The cell pellet was resuspended in 400 μl cold buffer A (10 mM Hepes, pH 7.9, 10 mM KCl, 0.1 mM EDTA, 0.1 mM EGTA, 1 mM DTT, 0.5 mM PMSF). The cells were allowed to swell on ice for 15 min, then 25 μl 10% NP-40 was added, and the tube was vortexed vigorously for 10 s. The homogenate was centrifuged at 10,000 g for 30 s. The nuclear pellet was resuspended in 50 μl ice-cold buffer B (20 mM Hepes, pH 7.9, 0.4 M NaCl, 1 mM EDTA, 1 mM EGTA, 1 mM DTT, 1 mM PMSF). After vigorously shaking at 4 °C for 15 min, the nuclear extracts were centrifuged at 10,000 g for 5 min at 4 °C, and the supernatant was collected. The protein concentrations were determined by Bradford. EMSA was performed using nuclear extracts from rat H9C2 myogenic cells according to the manufacturer’s instructions (Chemiluminescent Nucleic Acid Detection Module, Pierce). For super-shift, HIF-1α antibody was incubated with nuclear extracts for 1 h at 4 °C prior to adding the HRP (horseradish peroxidase)-labeled oligonucleotide. Wild-type probe (5′-TTCTTTCTCACGTGGCCATCAT-3′) and mutant probe (5′-TTCTTTCGAGCTTGGCCATCAT-3′) were generated according to the sequences of HRE site within the promoter of rat Mipu1.

### Luciferase reporter gene assay

The assay was performed according to the instruction of Dual Luciferase Reporter System (Promega) and the methods described previously [16-18]. Mipu1 promoter (-1170/+233) was cloned into PGL3 reporter vector as described previously in our lab [16]. H9C2 cells were seeded in 24-well plate. PGL3-Mipu1 reporter vector and pcDNA3.1-HIF-1α were co-transfected into cells for 12 h. All transfections were performed in triplicate from at least three independent experiments. 

### Expression of Results and Statistical Analysis

Data are represented as means ± S.E.M. of the number of independent experiment indicated (n) or as examples of representative experiments performed on at least three separate occasions. The two-tailed Student’s *t* test was used to compare the means of normally distributed continuous variables. Values *P*< 0.05 were chose as the limit of statistical significance.

## Results

### Cobalt chloride induces expression of Mipu1 and HIF-1α

**Figure 1:**
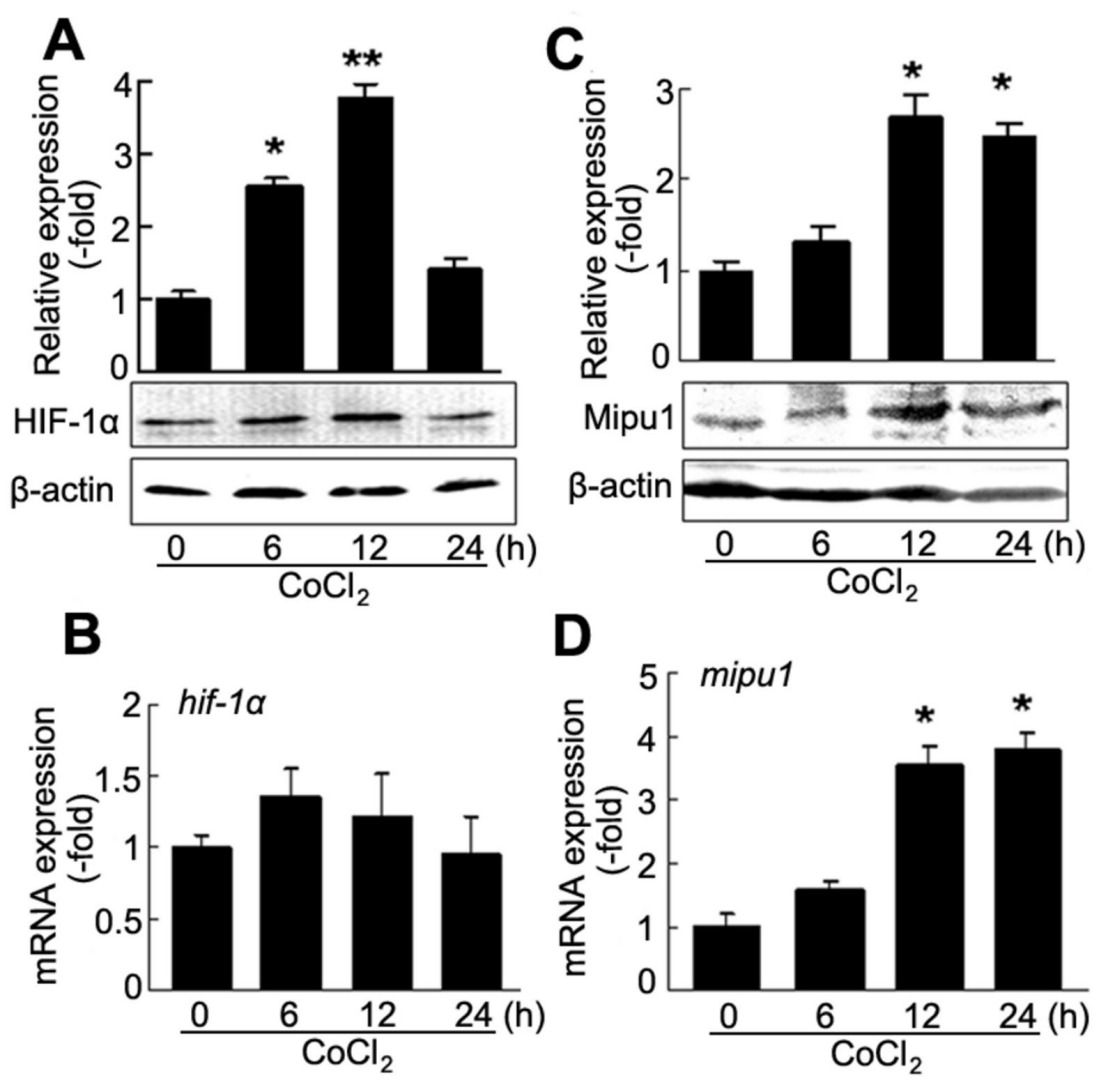
CoCl_2_ induces HIF-1α and Mipu1 expression in H9C2 cells. **A**: immunoblotting detection for CoCl_2_-induced expression of HIF-1α protein (n=3), densitometry analysis of HIF-1α band against β-actin band sown in upper panel. * *p*<0.05, ** *p*<0.01 versus “0” (CoCl_2_ untreated cells). **B**: Quantitative real time PCR detection for the expression of hif-1α induced by CoCl_2_ (n=4. triplicate for each sample). **C**: immunoblotting detection for CoCl_2_-induced expression of Mipu1 protein (n=3), densitometry analysis of Mipu1 band against β-actin band shown in upper panel. * *p*<0.05 versus “0” (CoCl_2_ untreated cells). **D**: Quantitative real time PCR detection for the expression of *mipu1* induced by CoCl_2_ (n=4. triplicate for each sample), compared with CoCl_2_ untreated cells, * *p*<0.05.

It has been demonstrated that HIF-1α is up-regulated in cobalt chloride (CoCl_2_)-mimicked hypoxic conditions *in vitro* cell model [23]. In our study, H9C2 cardiomyocytes were treated with 100 μM CoCl_2_. Western blotting showed that CoCl_2_ increased expression of HIF-1α at protein level and its expression peaked at 12 h (Figure 1A). However, CoCl_2_ failed to induce HIF-1α mRNA expression (Figure 1B). Since CoCl_2_ is an inhibitor of prolyl hydroxylases (PHDs) which hydroxylates HIF-1α protein at the proline residues leading to degradation of HIF-1α protein [24-26], it does make stable of HIF-1α protein rather than regulates the HIF-1α expression at transcriptional level. However, Mipu1 protein and mRNA expression were up-regulated at 12 h and lasted for 24 h after treatment with CoCl_2_ (Figure 1C and D). 

 Activation of HIF-1α is marked by its translocation from cytoplasm to nucleus under hypoxia [1-4]. Indirect immunofluoresence assay showed that CoCl_2_ increased accumulation of HIF-1α within the nucleus at 6 h (Figure S2 in File S1). These results revealed that CoCl_2_-induced expression and activation of HIF-1α were accompanied with an increase of Mipu1 protein and mRNA in H9C2 cells.

### HIF-1α regulates the expression of Mipu1

**Figure 2:**
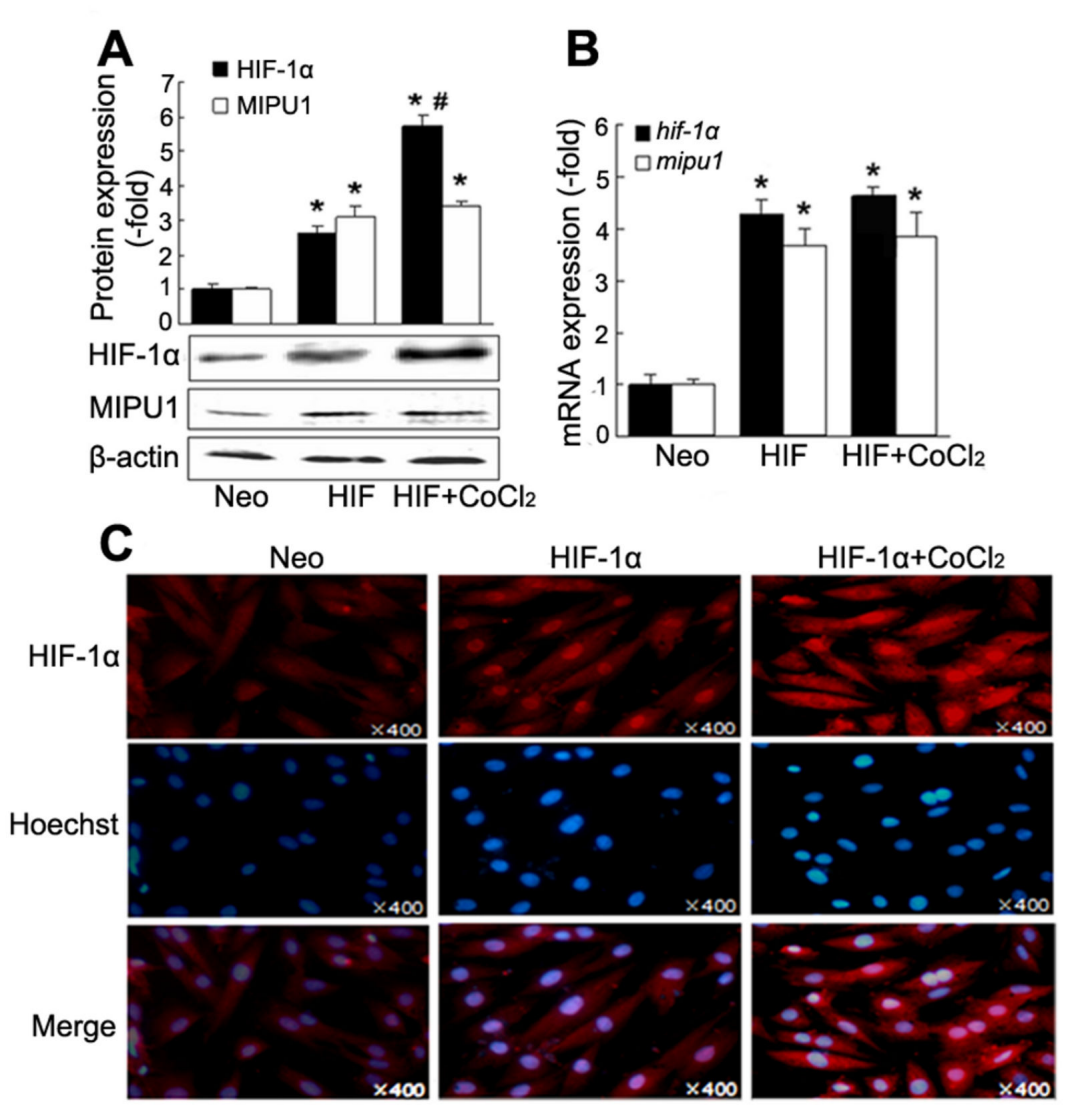
Over-expression of HIF-1α induces Mipu1 expression in H9C2 cells. **A**: immunoblotting showing the expression of Mipu1 and HIF-1α mediated by pcDNA3.1-HIF-1α transfection (n=3). Upper panel showing the semi-quantitative analysis of HIF-1α and Mipu1 band against β-actin. * *p*<0.05 versus “Neo” (pcDNA3.1 vector), # *p*<0.05 versus “HIF” (HIF-1α transfection alone). **B**: Quantitative real time PCR showing the expression of *mipu1* induced by HIF-1α transfection (n=3. triplicate for each sample), compared with “Neo”, * *p*<0.05. **C**: indirect immunofluorescence showing gene transfection-induced translocation of HIF-1α from cytoplasm to the nucleus (×400).

To elucidate if HIF-1α regulates the expression of Mipu1, HIF-1α plasmid was transfected into H9C2 cells and expression of Mipu1 was detected by Western blotting and real-time PCR. HIF-1 protein expression in cells treated with the combination of HIF-1α transfection and CoCl_2_ was more than that treated with HIF transfection alone, while no difference was observed for HIF-1α mRNA between the combination of HIF-1α transfection and CoCl_2_ and HIF-1α alone. The expression of Mipu1 both in protein and mRNA levels increased in cells transfected with HIF-1α plasmid. However, no difference was observed between the cells transfected with HIF-1α alone and the cells treated with HIF-1α transfection plus CoCl_2_ (Figure 2 A and B). The possibility is that the amount of HIF-1α protein induced by transfection is enough to induce the maximal expression of Mipu1. Immunofluoresense assay showed that activation of HIF-1α was induced by transfection with HIF-1α (Figure 2C). The results showed that HIF-1α activation promotes expression of Mipu1 in H9C2 cells.

**Figure 3:**
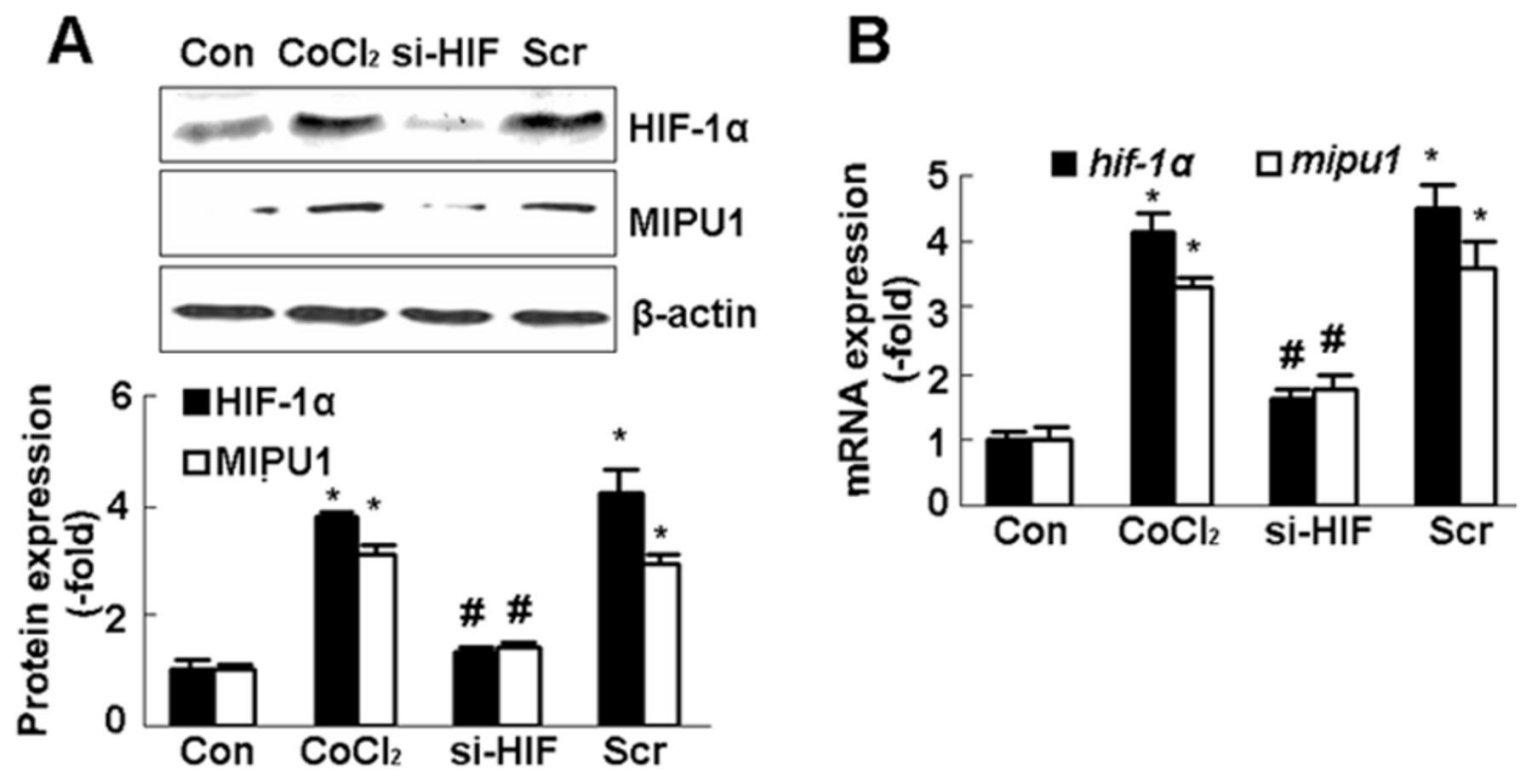
HIF-1α siRNA inhibits Mipu1 expression-induced by CoCl_2_ in H9C2 cells. **A:** immunoblotting showing the expression of Mipu1 and HIF-1α (n=3), the semi-quantitative analysis of HIF-1α and Mipu1 band against β-actin shown in bottom panel, “siHIF” and “Scr”: H9C2 cells were transfected with HIF-1α siRNA or scramble prior to CoCl_2_ stimulation. * *p*<0.05 versus “Con” (CoCl_2_ untreated cells), # *p*<0.05 versus “Scr” (scramble). **B:** Quantitative real time PCR showing the expression of *mipu1* induced by HIF-1α siRNA transfection (n=3. triplicate for each sample), * *p*<0.05 versus “Con” (CoCl_2_ untreated cells), # *p*<0.05 versus “Scr” (scramble).

 Next, we hypothesized that knock-down of HIF-1α would abolish the expression of Mipu1 induced by CoCl_2_. Prior to treatment with CoCl_2_ for 12 h, H9C2 cells were transfected with a specific small interference RNA (siRNA) oligomer against HIF-1α for 12 h. Western blotting and real-time PCR results showed that expression of HIF-1α protein and mRNA reduced to 40-50% of that induced by CoCl_2_, while, the protein and mRNA expression of Mipu1 decreased when HIF-1α was knocked-down (Figure 3). These results from gene gain-function and loss-function analysis indicated strongly that HIF-1α up-regulated Mipu1 expression. 

### HIF-1α binds to Mipu1 promoter

 It has been reported that under hypoxia conditions, stabilized HIF-1α translocates into nucleus and dimerizes with HIF-1β. Then the dimer binds to the HRE (5′-CACGTG-3′) motif in the promoter region to regulate the transcription of target genes [1-4,24,26,27]. By searching transcription factor binding sites with “TESS” and MatInspector software, we found that there is an HRE motif within the Mipu1 promoter region at positions -568 to -547 bp (Figure S1 in File S1). 

**Figure 4:**
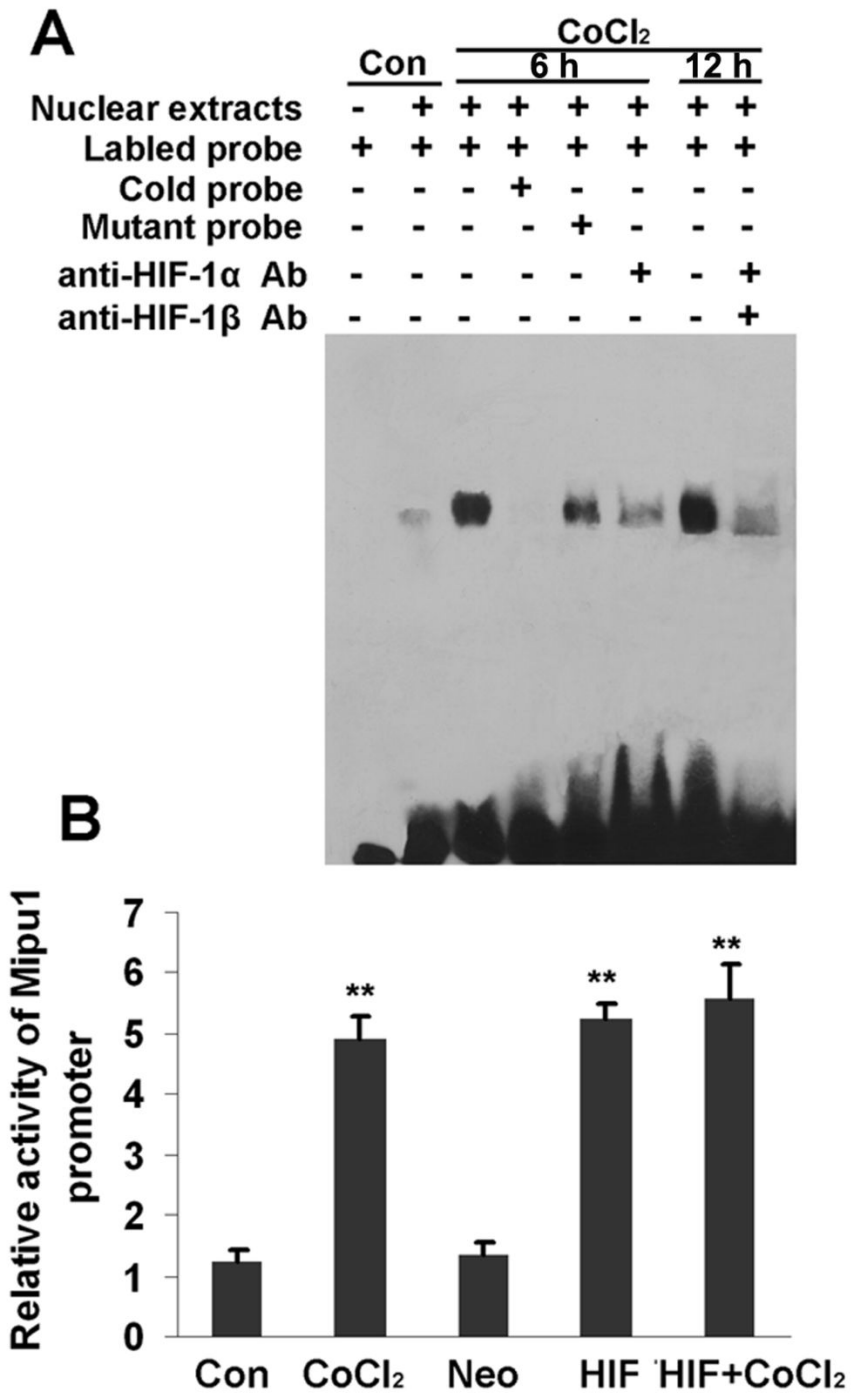
HIF-1α regulates the transactivation of Mipu1 promoter. **A**: EMSA showing HIF-1α binding with the HRE motif within the promoter region of Mipu1. “Con”: CoCl_2_-untreated control cells; “CoCl_2_”: cells treated with CoCl_2_ for 6 h and 12 h.** B:** fluorescence reporter gene analysis showing effects of HIF-1α and CoCl_2_ on the transactivation of Mipu1 promoter. “Con”: CoCl_2_-untreated control cells; “CoCl_2_”: cells treated with CoCl_2_ for 6 h, “HIF”: HIF-1α transfection for 12 h, “Neo”: pcDNA3.1 vector transfection for 12 h and “HIF+CoCl_2_”: HIF-1α transfection for 12 h prior to CoCl_2_ stimulation. ** *p*<0.01 versus “Con” and “Neo”.

 To test whether the HRE is enough for HIF-1α binding to the promoter of Mipu1 and therefore regulating the Mipu1 mRNA transcription, electrophoretic mobility shift assay (EMSA) was performed. Stronger binding activity was observed in nuclear extracts from CoCl_2_-treated H9C2 cells than that of control cells (Figure 4 A, Lane 3, Lane7 and lane 2). When the complexes of nuclear extracts and labeled probe were incubated with 100-fold unlabeled probe, the oligonucleotide binding activity was lost completely (Figure 4 A, Lane 4), and competitive effect of mutant probe was weak (Figure 4 A, Lane 5). Antibody-mediated super-shift assay showed that both anti-HIF-1α and anti-HIF-1β antibodies retarded the binding complex (Figure 4 A lane 6 and 8), indicating the binding of HIF-1α and β subunit with the specific oligonucleoltide probe.

### HIF-1 promotes the activity of reporter gene containing Mipu1 promoter

Here, we further examined the effect of HIF-1α on the activity of Mipu1 promoter by using luciferase reporter gene assay. For this experiment, the reporter plasmid of PGL-3-Mipu1 promoter (-1170/+233) was generated and transfected into H9C2 cells. After treatment with CoCl_2_ for 12 h, the expression of reporter gene was significantly increased in comparison with CoCl_2_-untreated cells (control, p<0.01). Over-expression of HIF-1α also increased the expression of reporter gene as well as CoCl_2_ treatment. However, the expression of reporter gene had no difference among the cells treated with CoCl_2_ alone, the cells transfected with HIF-1α alone and the cells treated with the combination of HIF-1α transfection and CoCl_2_. (Figure 4B). These results revealed that HIF-1α promoted the activity of Mipu1 promoter *in vivo*. These results from EMSA and reporter gene evidenced that Mipu1 is a novel direct target transcription factor downstream of HIF-1α.

### Mipu1 contributes to HIF-1-mediated cytoprotection

**Figure 5:**
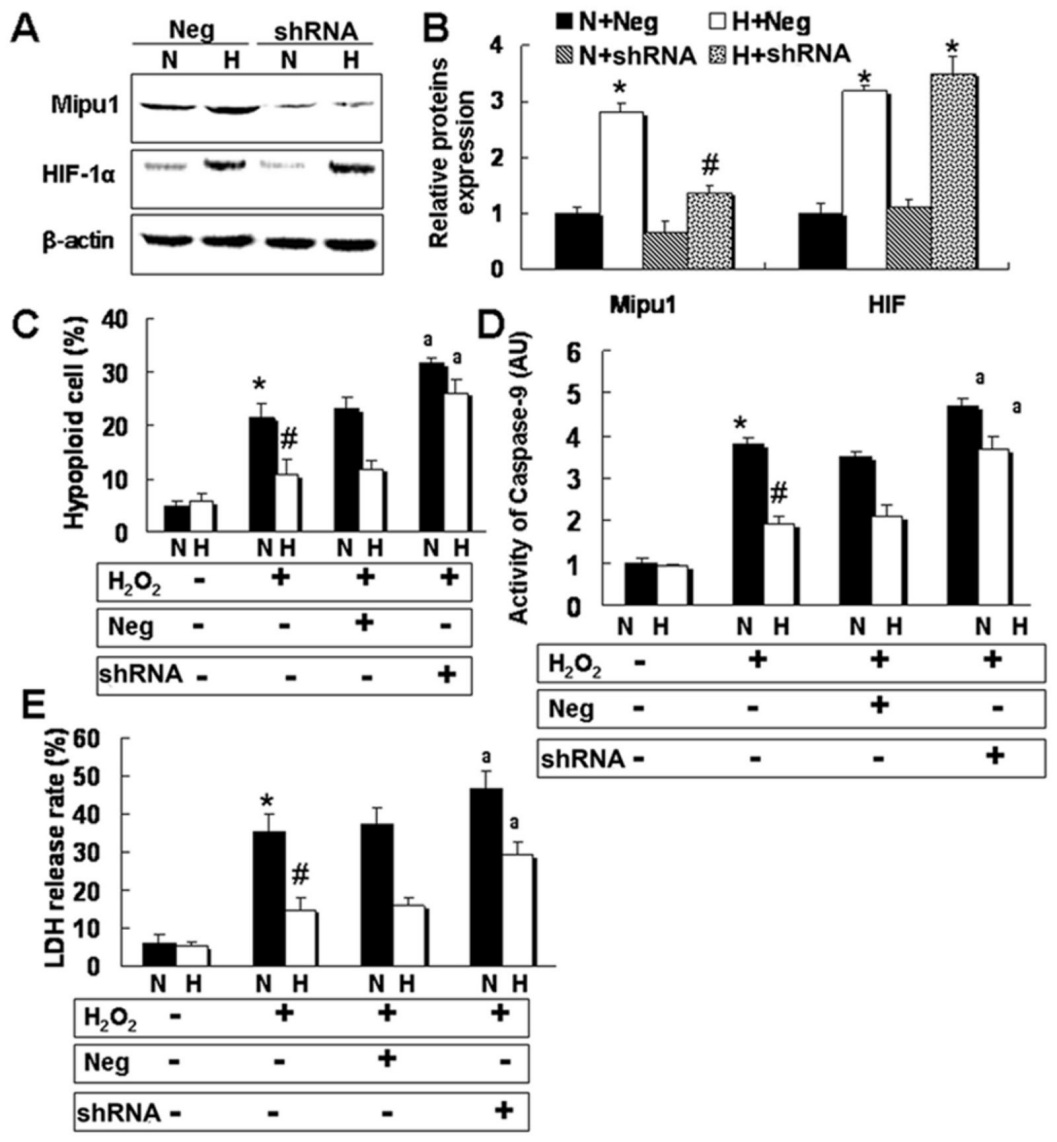
Mipu1 shRNA abolishes in part the cytoprotection of HIF-1α against oxidative stress in H9C2. “Neg”: negative control shRNA vector (pRNA-u6.1), “shRNA”: Mipu1 shRNA expression plasmid (pRNA-u6.1- Mipu1), “N”: pcDNA3.1 vector (Neo), “H”: pcDNA3.1-HIF-1α vector. **A**: immunoblotting showing the effect of Mipu1 shRNA on expression of Mipu1 and HIF-1α; **B:** semi-quantitative analysis of HIF-1α and Mipu1 band against β-actin, * *p*<0.05 versus “N+Neg”, # *p*<0.05 versus “H+Neg”, (n=3);** C:** flowcytometry detecting the hypoploid cells induced by 0.5 mmol/L hydrogen peroxide (H_2_O_2_), * *p*<0.05 versus H_2_O_2_-untreated cells, # *p*<0.05 versus H_2_O_2_-treated “N” group, a *p*<0.05 versus responsive “Neg” group, (N=5). **D**: Colorimetric Assay showing the activity of Caspase-9 induced by H_2_O_2_ (n=4). * *p*<0.05 versus H_2_O_2_-untreated cells, # *p*<0.05 versus H_2_O_2_-treated “N” group, a *p*<0.05 versus responsive “Neg” group. **E**: LDH release rate, * *p*<0.05 versus H_2_O_2_-untreated cells, # *p*<0.05 versus H_2_O_2_-treated “N” group, a *p*<0.05 versus responsive “Neg” group, n=5.

To further understand whether Mipu1 is involved in the cytoprotection of HIF-1 against oxidative stress, a plasmid containing short hair RNA (shRNA) against Mipu1 was delivered into H9C2 cells prior to HIF-1α transfection. Then, these co-transfected cells were treated with 0.5 mmol/L hydrogen peroxide (H_2_O_2_) as shown in our previous studies [20,28,29]. Results showed that shRNA mediated a significant decrease of the expression of Mipu1 protein and mRNA (Figure 5 A and B), whereas, it had no effect on the expression of HIF-1α and β-actin. Moreover, our results revealed that Mipu1 shRNA significantly abolished the protective effect of HIF-1 against cell injury induced by H_2_O_2_, as indicated by hypoploid cell numbers (Figure 5 C), the activity of caspase-9 (Figure 5 D) and release of lactate dehydrogenases (LDH) (Figure 5 E). 

**Figure 6:**
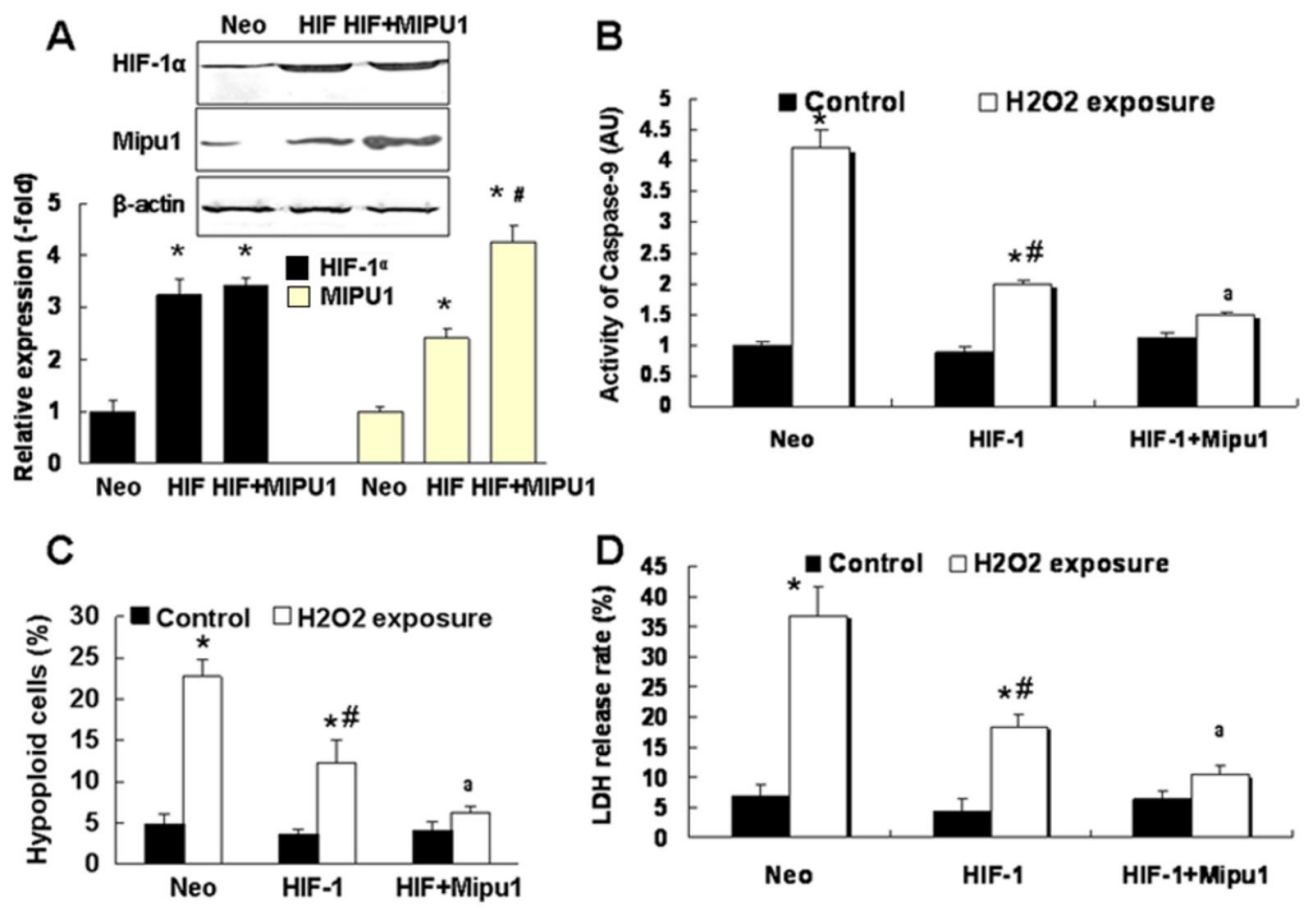
Mipu1 over-expression enhances the cytoprotection of HIF-1α against oxidative stress in H9C2 cells. “Neo”: pcDNA3.1 vector transfection group; “HIF”: pcDNA3.1-HIF-1α plasmid tranfection; “HIF+Mipu1”: cells cotransfected with pcDNA31-HIF-1α and pcDNA31-Mipu1 plasmids. **A**: immunoblotting showing the expression of Mipu1 and HIF-1α (n=3), bottom panel showing the semi-quantitative analysis of the Mipu1 and HIF-1α band against β-actin, * *p*<0.05 versus “Neo”, # *p*<0.05 versus “HIF” group. **B**: Colorimetric Assay showing the activity of Caspase-9 (n=4). * *p<0.05* versus H_2_O_2_-untreated cells, # *p*<0.05 versus H_2_O_2_-treated “Neo” group, a *p<0.05* versus H_2_O_2_-treated “HIF” alone group; **C**: flowcytometry detecting the hypoploid cells, * * *p*<0.05 versus H_2_O_2_-untreated cells, # *p*<0.05 versus H_2_O_2_-treated “Neo” group, a *p*<0.05 versus H_2_O_2_-treated “HIF” group, (N=5);** D:** LDH release rate, * *p*<0.05 versus H_2_O_2_-untreated cells, # *p*<0.05 versus H_2_O_2_-treated “Neo”group, a *p*<0.05 versus H_2_O_2_-treated “HIF” group, n=5.

Additionally, transfection with Mipu1 recombinant plasmid significantly increased the Mipu1 expression at protein and mRNA levels in HIF-1α-overexpressed H9C2 cells (Figure 6 A). After exposure to H_2_O_2_, cells co-transfected with Mipu1 and HIF-1α plasmids were characterized with lower hypoploid cells, activity of caspase-9 and LDH release rate than that of cells transfected with HIF-1ɑ alone (Figure 6 B-D). These results suggested strongly that Mipu1 contributed to the cytoprotection of HIF-1 against oxidative stress in H9C2 cells.

### Mipu1 down-regulates Bax expression involving in HIF-1-mediated cytoprotection

**Figure 7:**
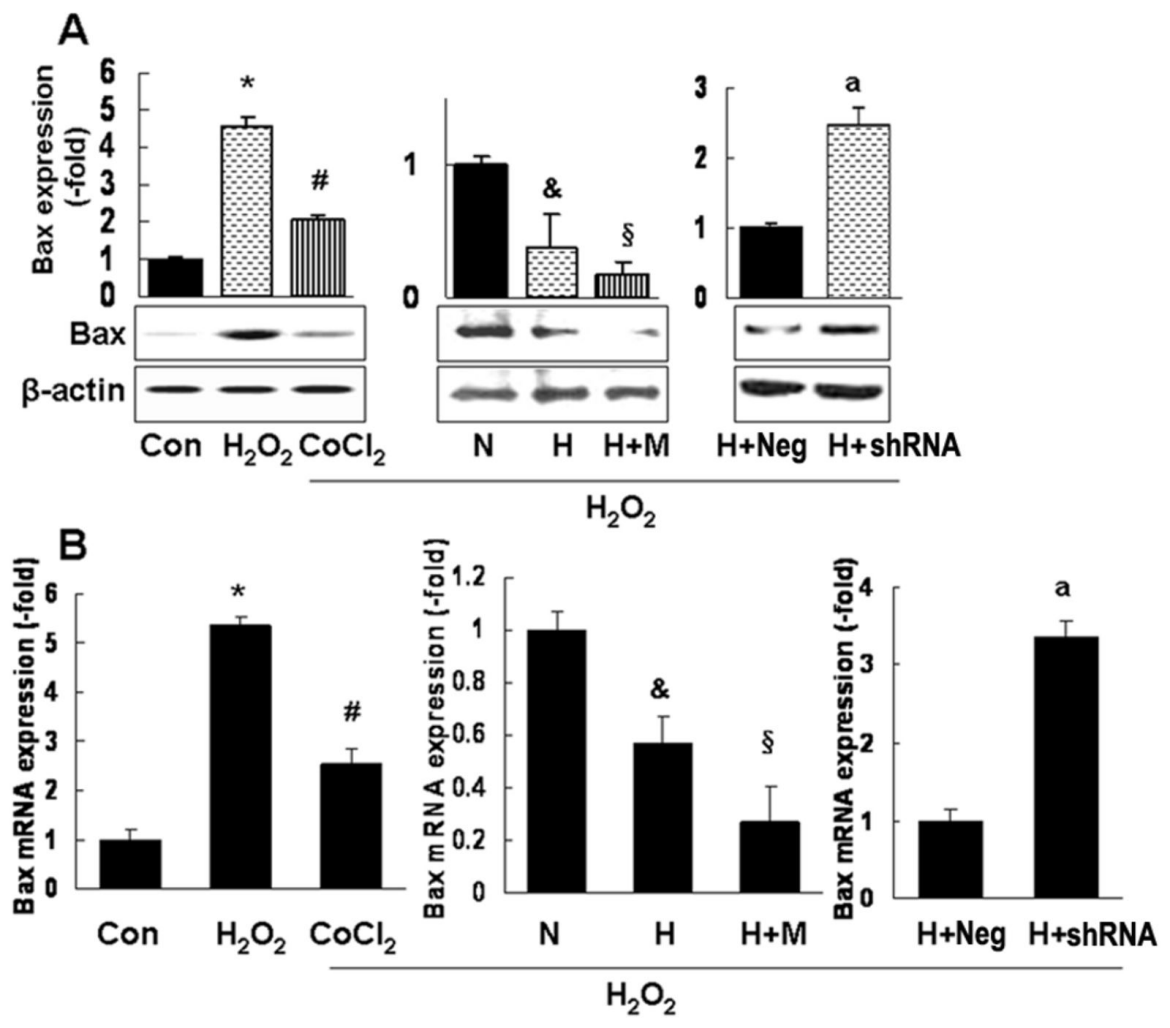
Mipu1 increases the inhibitory effects of HIF-1α on the expression of pro-apoptotic protein Bax in H9C2 cells. “Con”: H_2_O_2_-untreated control cell group; “H_2_O_2_”: H_2_O_2_-treated control cell group; “CoCl_2_”: cells pretreated with CoCl_2_ prior to H_2_O_2_ exposure; “N”: cells transfected with pcDNA3.1 control vector prior to H_2_O_2_ exposure; “H”: cells transfected with pcDNA3.1-HIF-1α plasmid prior to H_2_O_2_ exposure; “H+M”: cells cotransfected with pcDNA3.1-HIF-1α and Mipu1 plasmids prior to H_2_O_2_ exposure; “H+Neg”: cells cotransfected with pcDNA3.1-HIF-1α and pRNA-u6.1 plasmids prior to H_2_O_2_ exposure; “H+shRNA”: cells cotransfected with pcDNA3.1-HIF-1α and pRNA-u6.1-Mipu1-shRNA plasmids prior to H_2_O_2_ exposure. **A**: immunoblotting showing the Bax expression (n=3), upper panel showing the semi-quantitative analysis of Bax band against β-actin;** B:** quantitative real time PCR showing Bax mRNA expression (n=3, triplicate for each sample). Both in A and B: * *p*<0.05 versus control, “Con”, # *p*<0.05 versus “H_2_O_2_”, & *p*<0.05 versus “N”-, § *p*<0.05 versus “H”, a *p*<0.05 versus “H+Neg”. .

How does Mipu1 protect H_2_O_2_-injured cells? There are 6 potential Mipu1 binding elements core sequence (“CTTA”) within the promoter region of Bax (Figure S3 in File S1). We further found that H_2_O_2_ increased the expression of Bax at both protein and mRNA levels, whereas, CoCl_2_ pretreatment or HIF-1ɑ delivery remarkably inhibited its expression. And there was more reduction of Bax expression in cells co-transfected with Mipu1 and HIF-1ɑ plasmids than cells transfected with HIF-1ɑ plasmid alone. In contrast, when cells were co-transfected with Mipu1 shRNA and HIF-1ɑ plasmid, the Bax expression is elevated (Figure 7 and Figure S4 in File S1). It has been documented that Bax and caspase-9 are important pro-apoptotic gene associated with the mitochondrial apoptotic signal pathway [30,31]. In this study, both the down-regulation of Bax (as shown in Figure 7 and Figure S4 in File S1) and reduction of caspase-9 activity (as shown in Figure 5 and 6) were consistently observed in Mipu1-overexpressed cells. These results suggested that Bax might be a potential target gene of Mipu1, and that Mipu1 contributed partially to HIF-1ɑ-mediated cytoprotection via inhibiting Bax expression and activation of mitochondrial apoptotic pathway. 

## Discussion

 Mipu1, one of novel rat genes, was identified recently in our lab by using cDNA microarray and suppression subtractive hybridization [12]. A search of the rat genome sequence indicates that Mipu1 is composed of five exons and four introns and maps to chromosome 1q12.1. The molecular weight of rat Mipu1 protein is about 70 kDa with 608 amino acid residues containing a KRAB domain and 14 C2H2-type zinc finger domains. Its amino acid sequence and functional domain are highly consensus among mouse, human and rat (Figure S5 and S6 in File S1). 

 Our previous reports evidenced that Mipu1 was up-regulated in rat heart following a transient myocardial ischemia-reperfusion procedure and exhibited cytoprotection against oxidative stress and serum deprival [12,13,15]. However, its biological functions and regulatory expression are still not well documented. Here, we just focused on the mechanisms by which its expression is regulated at transcriptional level. 

 Previously, we cloned the full length promoter of Mipu1 by using 5′-Rapid amplification of cDNA ends (5′-RACE) and primer-extension and found that two GC boxes in close proximity to the transcription start site in Mipu1 promoter region were involved in the regulation of constitutive expression of Mipu1 [18]. Recently, we also found that CREB contributed to the inducible expression of Mipu1 mediated by H_2_O_2_ [19]. 

 Mipu1 was found and termed because of its upregulation mediated by ischemic preconditioning. It is well known that HIF-1 is a key transcription factor involving in hypoxia. Thus, we postulated that Mipu1 expression would be regulated by HIF-1. By using bioinformatic analysis, we found that there is an HIF-1 binding element (hypoxia response element) within the region of Mipu1 promoter. This study was designed to verify the possibility of HIF-1-mediated inducible expression of Mipu1. 

HIF-1 is a heterodimer composed of α and β subunits. Under normal oxygen levels, HIF-1α is unstable because PHDs use molecular oxygen as a substrate to hydroxylate proline residues of HIF-1α. The hydroxylated HIF-1α is recognized by the von Hippel-Lindau tumor suppressor (VHL), and targeted for proteasomal degradation [24,26,32]. It has been demonstrated that CoCl_2_ is an effective inducer of chemical hypoxia by inhibiting the activity of PHDs and preventing degradation of HIF-1α protein [25,33]. To establish a hypoxic cellular model, CoCl_2_ was employed to mimic the hypoxic condition in this study. We found that Mipu1 is a target gene of HIF-1α, the latter translocates into nucleus and binds to the HRE motif within the promoter region of Mipu1 and activates its transcription. 

It has been reported previously that HIF-1 serves as an important endogenous cytoprotective gene by which it maintains oxygen homeostasis via inducing expression of a cluster genes, such as EPO, HO-1 and iNOS, at transcriptional level [5-7,33,34]. Recently, Manalo Dj et al [11] reported that HIF-1 can regulate the expression of 26 transcription factors in human pulmonary artery endothelial cell, for example myocyte enhancer factor 2A (MEF2A), peroxisome proliferative activated receptor gamma (*PPAR-γ*), zinc finger protein 292 (ZNF292), nuclear factor of activated T cells 4 (NFATc4), cAMP responsive element binding protein 3-like 2 (*CREB3L2*) and so on. In this study, we reported that HIF-1 exerted its cytoprotection against oxidative stress-induced cell injury partially by promoting expression of a novel transcription factor Mipu1 in H9C2 cells.

 Previously, our lab reported that H_2_O_2_ (0.5 mmol/L) induced apoptotic cell death rather than necrotic cell death by activating the mitochondrial apoptotic pathway in H9C2, C2C12 and human umbilical vein endothelial cell lines [20,28,29]. Caspase-9 is an important apoptotic protease downstream of mitochondrion. Death stimuli (for example H_2_O_2_) open the mitochondrial permeability transition pore (mPTP) and lead to release of proapoptotic factors (Cyt c, Apaf-1, Smac/DIABLO, and so on). Cyt C and Apaf-1 bind to and activate caspase-9, subsequently induce apoptosome [31,35]. In this study, we first observed that HIF and Mipu1 inhibited the H_2_O_2_-mediated apoptosis by decreaseing the activity of caspase-9. These results suggested that Mipu1 may be a transcriptional factor regulating the mitochondrial apoptotic pathway. 

However, it is unclear how Mipu1 regulates mitochondrial function. It has been documented that the mitochondrial apoptotic pathway is controlled by the members of Bcl-2 family (for example Bax, Bak, Bik, Bcl-2, Bcl-XL and so on). Bax is a major final mediator of the outer mitochondrial membrane (OMM) permeabilization. When Bax punctures the OMM, the mPTP is opened and the mitochondrial contents release into cytoplasm [30,31,36]. Our previous studies have reported that H_2_O_2_ could increase the Bax expression [37,38]. In this study, we analyzed the promoter of *bax* gene and found that there are 6 motifs of core sequence (“CTTA”) of Mipu1 binding element (Figure S3 in File S1). So, we hypothesized that Mipu1 regulates the mitochondrial apoptotic pathway by controlling the expression of Bax at transcriptional level. In this study, our primary results showed that Mipu1 inhibited the protein and mRNA expression of Bax induced by H_2_O_2_ exposure in H9C2 cells. The consistent results between Bax expression and caspase-9 activity mediated by Mipu1 overexpression or down-regulation suggested that Mipu1 might regulate mitochondrial apoptotic pathway by controlling the *bax* transcription and Bax could be the potential direct target of Mipu1. Further studies need to be done for evidencing Mipu1-mediated regulation of Bax expression at transcriptional level. 

Taken together, our results suggested that Mipu1 is involved in HIF-1-mediate cytoprotection by inhibiting the expression of Bax and interfering activation of mitochondrial apoptotic pathway (Figure S7 in File S1). Our findings provided a novel insight for understanding the cytoprotection role of HIF-1α and provide new clues for further elucidating the mechanisms by which Mipu1 protects cell against pathological stress.

## Supporting Information

File S1 **Contains Figures S1-S7.** Figure S1. Sequence of the promoter region and 5’-UTR of Mipu1 gene. Partial sequence (-1170/+233) of the promoter region and 5’-UTR of Mipu1 gene was previously identified in our lab. A HRE element (-568/-547) was found by using MatInspector software (http://www.genomatix.de/online_help/help_matinspector/matinspector_help.html) and TESS software (http://www.cbil.upenn.edu/tess/). Figure S2. CoCl2 induces translocation of HIF-1α from cytoplasm to the nucleus in H9C2. As mentioned in Material and Methods, FITC-labeled secondary antibody was hybridized with anti-HIF-1α antibody. Hoechst 33258 was used for the counterstaining of the nucleus. Images were captured under Olympus BX61WI upright fluorescence microscope and were presented at 400× amplification. Figure S3. The 6 Mipu1 bindingcore sequence binding to within the promoter region of rat Bax gene (AB046392). The underlined words in red indicate the core sequences “CTTA” of Mipu1 binding element. Figure S4. Mipu1 shRNA attenuated the inhibitory effect of HIF-1α on the expression of Bax. A: immunoblotting showing the expression of Bax protein; B: Real time PCR showing the expression of Bax mRNA. “Neg” is the negative control (cells transfected with pRNA-u6.1 plasmids) against Mipu1 shRNA; “shRNA” means the cells transfected with pRNA-u6.1-Mipu1-shRNA plasmids. * p<0.05 versus control (Column 1), # p<0.05 versus Column 2, $ p<0.05 versus Column 4. Figure S5. Homologous comparisons of the amino acid (AA) sequence of Mipu1 among rat, mouse and human by using ClustalW software (http://www.ebi.ac.uk/Tools/msa/clustalw2/). The bottom table showed the score of identity of AA sequence. Figure S6. The conservative functional domains of Mipu1 protein among human, rat and mouse. The information was obtained by using the Prosite software (http://prosite.expasy.org/). All of 3 Mipu1 proteins have a KRAB domain and 14 (mouse and rat Mipu1) or 15 (human Mipu1) C2H2-type ZNF domain. KRAB: Krϋppel-associated box domain (green rectangle), ZNF: zinc finger domain (gray pentagon). Figure S7. The schematic diagram of the cytoprotection of HIF-1 and Mipu1 against oxidative stress (H2O2) mediated apoptosis by mitochondrial apoptotic pathway.
(DOC)
