# Supplementary figures and images for: Mipu1, a Novel Direct Target Gene, Is Involved in Hypoxia Inducible Factor 1-Mediated Cytoprotection
Source: PLoS One. 2013 Dec 11;8(12):e82827. doi: 10.1371/journal.pone.0082827 (PMC3859620; doi:10.1371/journal.pone.0082827)

**Figure S1.**

**Figure S2.**


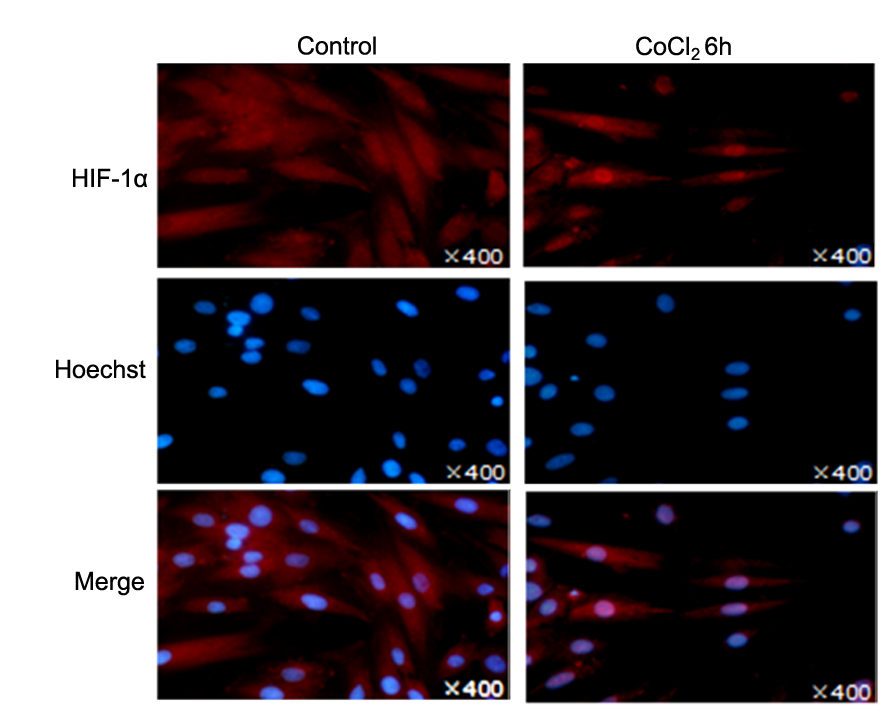


**Figure S3.**


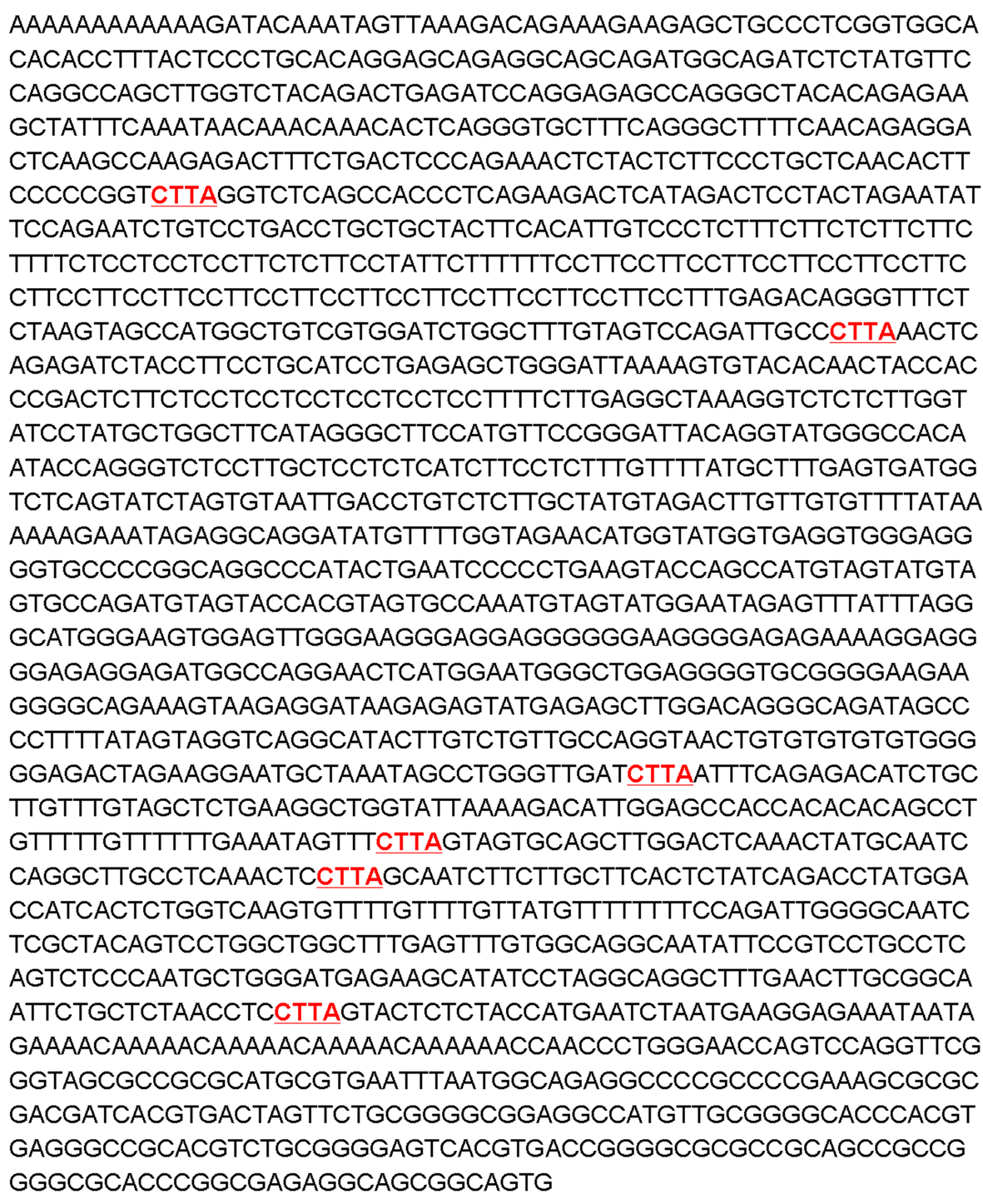


**Figure S4.**

**
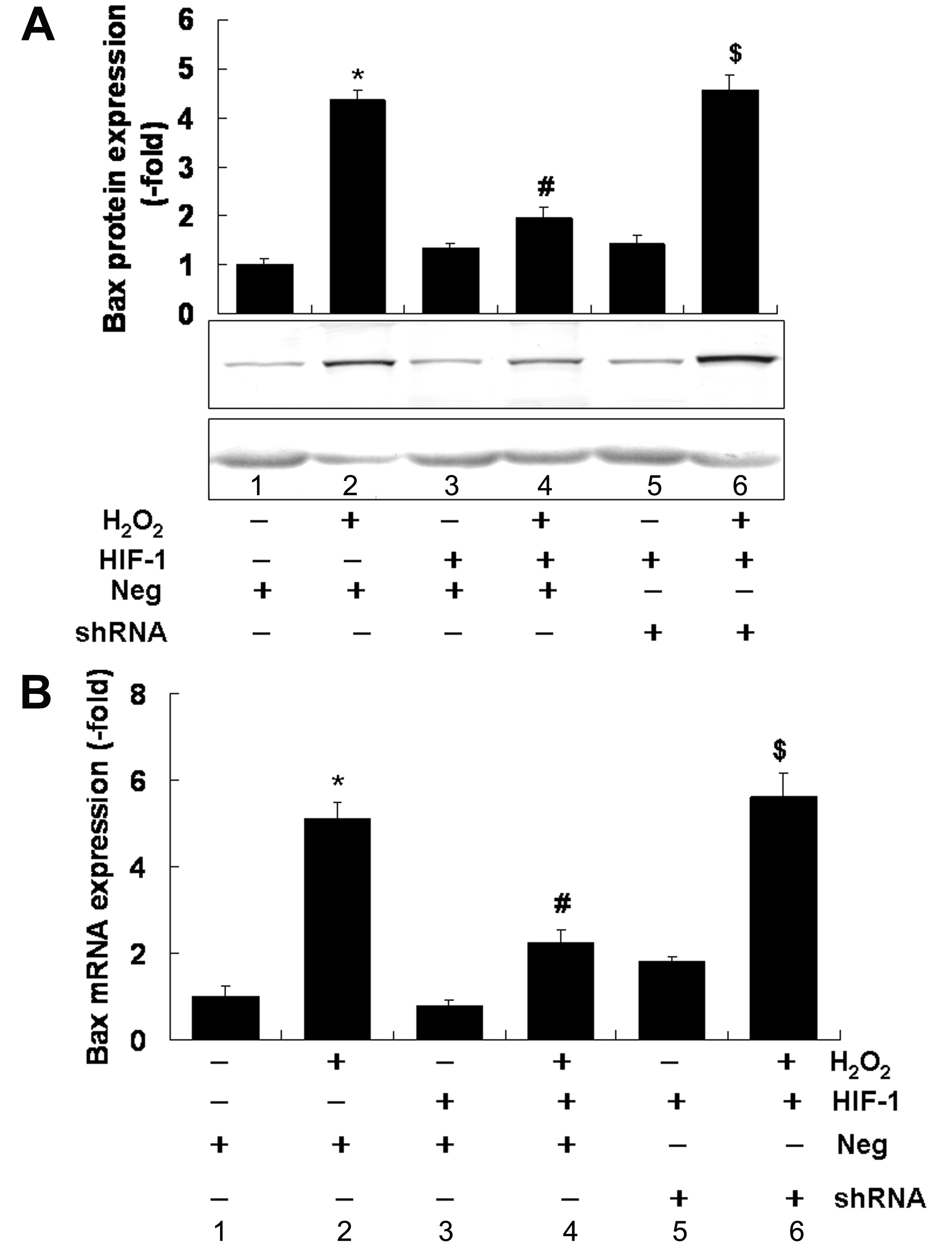
**

**Figure S5.**


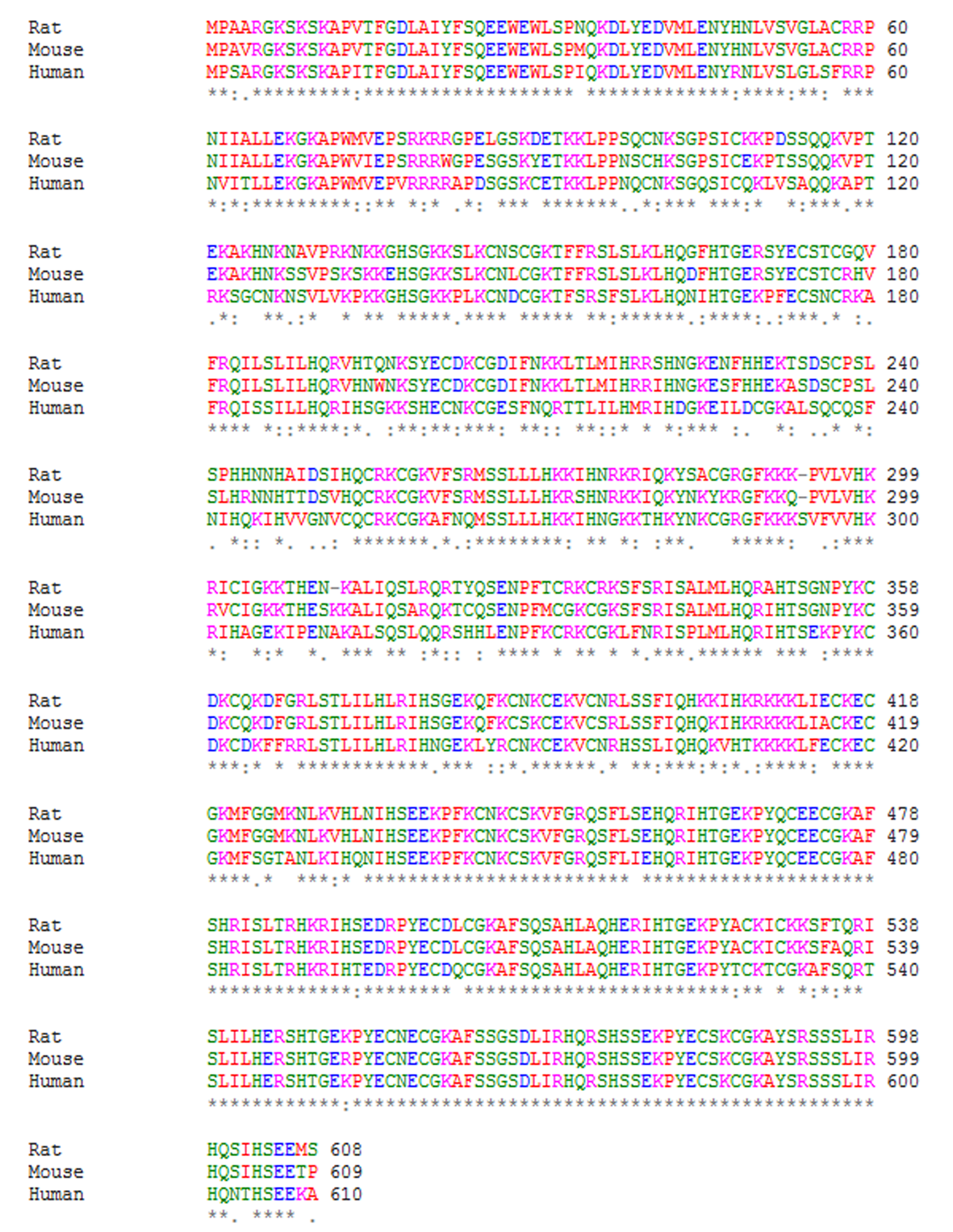


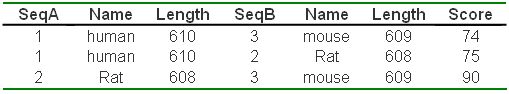


**Figure S6.**


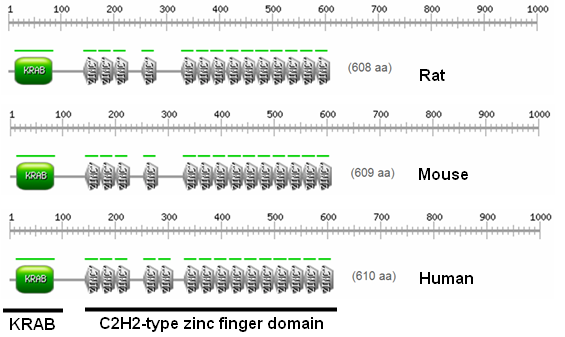


**Figure S7.**


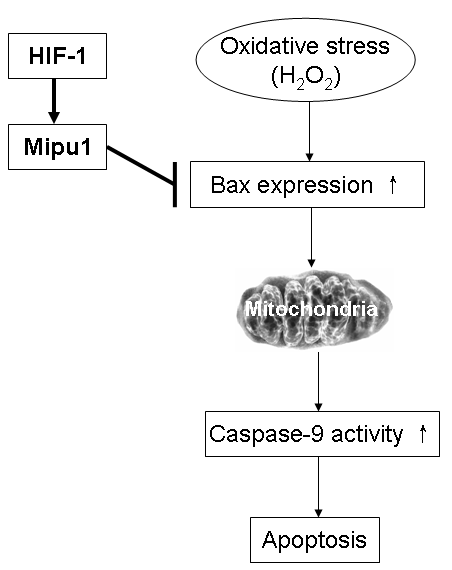

Supplement: File S1 — Contains Figures S1-S7. Figure S1. Sequence of the promoter region and 5’-UTR of Mipu1 gene. Partial sequence (-1170/+233) of the promoter region and 5’-UTR of Mipu1 gene was previously identified in our lab. A HRE element (-568/-547) was found by using MatInspector software (http://www.genomatix.de/online_help/help_matinspector/matinspector_help.html) and TESS software (http://www.cbil.upenn.edu/tess/). Figure S2. CoCl2 induces translocation of HIF-1α from cytoplasm to the nucleus in H9C2. As mentioned in Material and Methods, FITC-labeled secondary antibody was hybridized with anti-HIF-1α antibody. Hoechst 33258 was used for the counterstaining of the nucleus. Images were captured under Olympus BX61WI upright fluorescence microscope and were presented at 400× amplification. Figure S3. The 6 Mipu1 bindingcore sequence binding to within the promoter region of rat Bax gene (AB046392). The underlined words in red indicate the core sequences “CTTA” of Mipu1 binding element. Figure S4. Mipu1 shRNA attenuated the inhibitory effect of HIF-1α on the expression of Bax. A: immunoblotting showing the expression of Bax protein; B: Real time PCR showing the expression of Bax mRNA. “Neg” is the negative control (cells transfected with pRNA-u6.1 plasmids) against Mipu1 shRNA; “shRNA” means the cells transfected with pRNA-u6.1-Mipu1-shRNA plasmids. * p<0.05 versus control (Column 1), # p<0.05 versus Column 2, $ p<0.05 versus Column 4. Figure S5. Homologous comparisons of the amino acid (AA) sequence of Mipu1 among rat, mouse and human by using ClustalW software (http://www.ebi.ac.uk/Tools/msa/clustalw2/). The bottom table showed the score of identity of AA sequence. Figure S6. The conservative functional domains of Mipu1 protein among human, rat and mouse. The information was obtained by using the Prosite software (http://prosite.expasy.org/). All of 3 Mipu1 proteins have a KRAB domain and 14 (mouse and rat Mipu1) or 15 (human Mipu1) C2H2-type ZNF domain. KRAB: Krϋppel-associated [file pone.0082827.s001.doc]
